# A Drug-Target Network-Based Approach to Evaluate the Efficacy of Medicinal Plants for Type II Diabetes Mellitus

**DOI:** 10.1155/2013/203614

**Published:** 2013-10-10

**Authors:** Jiangyong Gu, Lirong Chen, Gu Yuan, Xiaojie Xu

**Affiliations:** Beijing National Laboratory for Molecular Sciences, State Key Lab of Rare Earth Material Chemistry and Applications, College of Chemistry and Molecular Engineering, Peking University, Beijing 100871, China

## Abstract

The use of plants as natural medicines in the treatment of type II diabetes mellitus (T2DM) has long been of special interest. In this work, we developed a docking score-weighted prediction model based on drug-target network to evaluate the efficacy of medicinal plants for T2DM. High throughput virtual screening from chemical library of natural products was adopted to calculate the binding affinity between natural products contained in medicinal plants and 33 T2DM-related proteins. The drug-target network was constructed according to the strength of the binding affinity if the molecular docking score satisfied the threshold. By linking the medicinal plant with T2DM through drug-target network, the model can predict the efficacy of natural products and medicinal plant for T2DM. Eighteen thousand nine hundred ninety-nine natural products and 1669 medicinal plants were predicted to be potentially bioactive.

## 1. Introduction

Type II Diabetes mellitus (T2DM) has been a major global health problem and affects a large population worldwide [1, 2]. T2DM is a multifactorial and genetically heterogeneous disease caused by various risk factors such as insulin resistance, *β*-cell dysfunction, and obesity [2–5]. Moreover, T2DM may cause acute cardiovascular disease, retinopathy, nephropathy, neuropathy, and kidney-related complications [5–7]. Therefore, it demands effective drugs with minimal toxicity. The herbal medicines have been used for T2DM for thousands of years and accumulated a great deal of clinical experience. A herbal formula comprises several medicinal plants or animals and thus can affect the biological system through interactions between compounds and cellular targets [3, 8–17]. The main mechanisms of herbal medicines in treating T2DM are that it increases insulin secretion and the sensitivity of insulin, inhibits glucose absorption, and reduces radicals caused by lipid peroxidation [8]. However, the major problem of herbal medicines is lack of scientific and clinical data to evaluate their efficacy and safety.

Network pharmacology proposed by Hopkins is a holistic approach to understand the function and behavior of a biological system at systems level in the context of biological networks and would be the next paradigm for drug discovery [18–20]. Several efforts have been made to explore the mechanism of herbal medicines such as prediction of the active ingredients and potential targets [21–26] and screening synergistic drug combinations [11, 27, 28]. The drug-target network (DTN) which connects drugs and their target proteins is an important biological network and provides an overview of polypharmacology of drugs [29–32]. Since medicinal plants have multiple compounds and a compound would have several target proteins, the DTN may bridge the gap between medicinal plants and diseases. In this work, we developed a computational approach based on DTN to evaluate the efficacy of medicinal plants.

## 2. Materials and Methods

### 2.1. Data Collection and Molecular Docking

**Figure 1:**
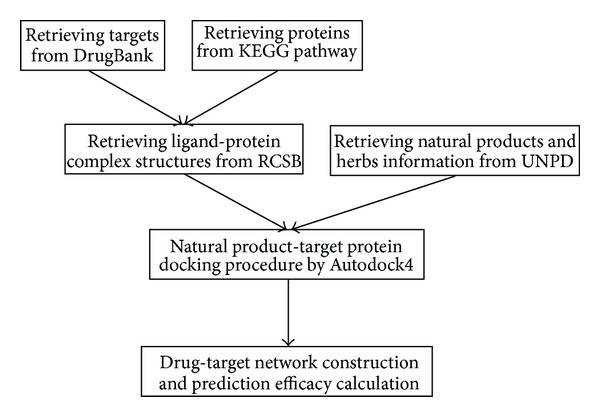
The work flow of this approach.

The pathogenesis of T2DM is concerned with various proteins. We retrieved the information of these proteins from KEGG Pathway database [33] and DrugBank [34] (Figure 1). The pathway of T2DM was downloaded from the KEGG website (http://www.genome.jp/dbget-bin/www_bget?hsa04930), and the information of T2DM-related proteins was collected. In DrugBank, we first retrieved the FDA-approved drugs for T2DM and then found the target proteins for each drug. Then we searched the ligand-protein complex structure (x-ray or NMR) for each protein from RCSB protein data bank (http://www.rcsb.org/pdb/home/home.do). Finally, thirty-three proteins and their information were listed in Table 1.

The 3D structures of natural products contained in medicinal plants were retrieved from the Universal Natural Product Database (UNPD) which comprised more than 208 thousands of natural products [54, 55]. The AutoDock 4.0 [56, 57] was adopted to perform the virtual screening, and binding free energy-based docking score (*pK*_*i*_) was used to evaluate the affinity between each compound and each protein. For each protein, the hetero atoms of the ligand-protein complex structure were deleted and the polar hydrogen atoms were added. The binding site of each protein was defined as a 40  ×  40  ×  40 Å cube around the original ligand with a spacing of 0.375 Å between the grid points. The center of binding site was located in the center of the original ligand. The molecular docking was conducted according to the protocol described previously [58].

### 2.2. Drug-Target Network Construction and Analysis

The drug-target network was constructed by linking the compound with target protein if the docking score satisfied the thresholds that were used to determine whether the interaction between compound and protein was strong. According to our previous study, the thresholds were set as follow: the docking score should be greater than 7.00 and the score of original ligand of corresponding protein and the top percentage of rank of docking score should be less than 10% [54]. The edge value was the docking score of corresponding compound and protein. Finally, the DTN consisted of 32 target proteins, 18999 compounds (the UNPD ID, chemical name, formula, molecular weight, and CAS registry number of each compound were listed in Table S1, see Table S1 in Supplementary Material available online at http://dx.doi.org/10.1155/2013/203614), and 35076 edges (Supplementary Table S2). The glucocorticoid receptor (P04150) did not have any compounds. The compounds were derived from 1669 medicinal plants distinguished by Latin names. The DTN of potentially active compounds and proteins related with T2DM was used as a bridge to build the relationship between compound or medicinal plant and T2DM.

### 2.3. Chemical Space Analysis

**Figure 2:**
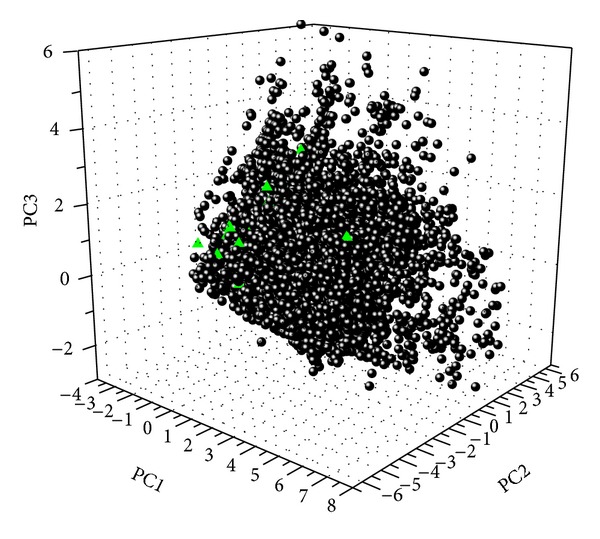
The distribution in chemical space according to PCA of natural products contained in medicinal plants and 25 FDA-approved drugs for T2DM. The black dots and green triangles represent natural products and FDA-approved drugs, respectively.

The analysis of the distribution of compounds in the chemical space was conducted by principal component analysis (PCA) module in Discovery Studio. The PCA model was built with 8 descriptors: *A*  log⁡  *P*, molecular weight, number of hydrogen-bond donors, number of hydrogen-bond acceptors, number of rotatable bonds, number of rings, number of aromatic rings, and molecular fractional polar surface area. The variances of PC1, PC2, and PC3 for compounds in Figure 2 were 0.488, 0.186, and 0.145, respectively. The PCA of 25 FDA-approved small-molecule drugs retrieved from DrugBank was performed in the same process as above.

### 2.4. Prediction Model

Natural products are multitarget agents. The average number of target proteins was 1.84 in the DTN. Therefore, we proposed that the prediction efficacy (PE) of a compound for T2DM was the sum of its all edge values (docking scores) in the DTN:

(1) $$\begin{array}{c} \text{P}{\text{E}}_{\text{compound}}=\overset{}{\underset{j\in P}{\sum }}\text{scor}{\text{e}}_{j}, \end{array}$$

where *P* was the set of proteins related to T2DM and score_*j*_ was the docking score between this compound and *j*th protein. The PE_compound_ for each compound was listed in Table S3.

Similarly, the prediction efficacy of a medicinal plant was defined as the sum of PE of compounds contained in this plant:

(2) $$\begin{array}{c} \text{P}{\text{E}}_{\text{plant}}=\overset{N}{\underset{i}{\sum }}\text{P}{\text{E}}_{{\text{compound}}_{i}}, \end{array}$$

where *N* denoted the number of compounds contained in the medicinal plant. The PE_plant_ for each medicinal plant was listed in Table S4.

## 3. Results and Discussion

### 3.1. Drug-Likeness of Medicinal Natural Products for T2DM

The natural products contained in medicinal plants for T2DM had good drug-like properties. Lipinski CA and colleagues proposed the “rule of five” (molecular weight (MW) less than 500 Da, the number of hydrogen bond acceptors (HBA) less than 10, the number of hydrogen bond donors (HBD) less than 5, and octanol-water partition coefficient (*A*  log⁡  *P*) less than five) [59, 60] to estimate solubility and permeability of compounds in drug discovery. That is, a compound was unlikely to be a drug if it disobeyed the rules. The mean and median of MW, HBA, HBD, and *A*  log⁡  *P* of these compounds were 540.43, 494.62; 6.3, 5; 2.5, 2; and 4.94, 5.07; respectively. It indicated that most compounds would be drug-like. The wide distribution of natural products in chemical space (Figure 2) showed that there would be vast property (structural and functional) diversity. Moreover, the large overlap between natural products and 25 FDA-approved small-molecule drugs for T2DM demonstrated that natural products contained in these medicinal plants had a hopeful prospect for drug discovery for T2DM.

### 3.2. Prediction Efficacy of Natural Product and Medicinal Plant

Herb medicines could simultaneously target multiple physiological processes through interactions between multiple compounds and cellular target proteins. For example, there were 105 distinct compounds contained in *Hypericum perforatum*, and 21 compounds existed in DTN. The herbal medicines could influence the biological system through interactions between multi-component and multi-target and thus reverse the biological networks from disease state to health state. Since a group of compounds contained in the herbal medicine could play a therapeutic role, the dosage could be reduced to reduce toxicity and side effects. For example, UNPD43323 (ormojine), UNPD194973 (ormosinin), and UNPD194973 (strychnohexamine) were the top three potential compounds (Supplementary Table S3). ormojine, ormosinin, and strychnohexamine had 27, 24, and 23 targets, respectively. The polypharmacology of natural products was very common.

The predicted efficacy of the top twenty medicinal plants for T2DM was listed in Table 2. There were five plants (*Hypericum perforatum, Ganoderma lucidum, Holarrhena antidysenterica, Celastrus orbiculatus, *and* Murraya euchrestifolia*) where prediction efficacy was higher than 1000. We searched the literatures which reported the anti-T2DM bioactivities of the top twenty medicinal plants (Table 2) and found that 15 medicinal plants had information of definite effectiveness against T2DM. For example, Arokiyaraj and colleagues evaluated the antihyperglycemic activity of *Hypericum perforatum* in diabetic rats, and it produced significant reduction in plasma glucose level [35].

### 3.3. Clinical Herbal Formula

Tangminling which was a widely used herbal formula in China to treat T2DM comprised eleven medicinal herbs (*Trichosanthes kirilowii*, *Citrus sinensis*, *Bupleurum chinense*, *Rheum officinale*, *Astragalus membranaceus*, *Pinellia ternata*, *Scutellaria discolor*, *Crataegus pinnatifida var*. *major*, *Paeonia albiflora*, *Prunus mume*, and *Picrorhiza kurroa*) [3]. The prediction efficacy of each medicinal plant was 493.04, 199.26, 36.06, 29.08, 15.12, 14.80, 7.83, 7.09, 7.07, 7.06, and 7.04, respectively. It indicated that all plants could play a role in the treatment of T2DM. However, the prediction efficacy of eleven herbs differed considerably from each other. It meant that *Trichosanthes kirilowii* and *Citrus sinensis* played major roles (sovereign herbs). Meanwhile, The others worked as assistants which may strengthen the efficacy of sovereign herbs or reduce the toxicity.

## 4. Conclusions

Medicinal plants are potentially important for novel therapeutic drugs. It is currently estimated that approximately 420,000 plant species exist in nature [61]. However, only 10,000 of all plants have documented medicinal use [62]. Therefore, there are potentially many more important pharmaceutical applications of plants to be exploited. Traditional method (from selecting plants to separating compounds following bioassay) is time-consuming. In this work, we developed a molecular docking score-weighted prediction model based on drug-target network to evaluate the efficacy of natural products and medicinal plants for T2DM. Natural products contained in the medicinal plants would target several cellular target proteins. The prediction efficacy of this model took into account all potential interactions between multicomponents and targets. Therefore, the prediction efficacy was an overall evaluation at systems level. Fifteen out of the top twenty medicinal plants had reported bioactivity against T2DM in literatures. This approach may promote the research on the use of medicinal plants to treat T2DM and drug discovery from natural products.

## Supplementary Material

The supplementary materials comprise four tables of large datasets. Table S1 listed the identification information of 18999 natural products. Table S2 listed the natural products-target proteins interaction network (DTN). Table S3 and Table S4 listed the prediction efficacy of natural products and medicinal plants for T2DM, respectively.

## Figures and Tables

**Table 1:** List of 33 proteins related with T2DM for molecular docking.

Index	UniProt entry	PDB entry	Protein name
1	O43451	3CTT	Maltase-glucoamylase, intestinal
2	P01308	1TYM	Insulin
3	P01375	2AZ5	Tumor necrosis factor alpha
4	P04150	3H52	Glucocorticoid receptor
5	P04746	1XDO	Pancreatic alpha-amylase
6	P05121	3UT3	Plasminogen activator inhibitor 1
7	P06213	3EKN	Insulin receptor
8	P07339	1LYW	Cathepsin D
9	P08069	3I81	Insulin-like growth factor 1 receptor
10	P11474	3K6P	Steroid hormone receptor ERR1
11	P12821	3L3N	Angiotensin-converting enzyme
12	P13569	3GD7	Cystic fibrosis transmembrane conductance regulator
13	P14410	3LPP	Sucrase-isomaltase, intestinal
14	P14618	3BJF	Pyruvate kinase isozymes M1/M2
15	P14735	3E4A	Insulin-degrading enzyme
16	P19367	1DGK	Hexokinase-1
17	P27361	2ZOQ	Mitogen-activated protein kinase 3
18	P27487	3G0D	Dipeptidyl peptidase 4
19	P27986	4A55	Phosphatidylinositol 3-kinase regulatory subunit alpha
20	P28482	3I5Z	Mitogen-activated protein kinase 1
21	P30613	2VGF	Pyruvate kinase isozymes R/L
22	P35557	3IMX	Glucokinase
23	P35568	2Z8C	Insulin receptor substrate 1
24	P37231	3H0A	Peroxisome proliferator-activated receptor gamma
25	P42336	3HHM	Phosphatidylinositol 4,5-bisphosphate 3-kinase catalytic subunit alpha isoform
26	P42345	1FAP	Serine/threonine-protein kinase mTOR
27	P43220	3C59	Glucagon-like peptide 1 receptor
28	P45983	3PZE	Mitogen-activated protein kinase 8
29	P45984	3NPC	Mitogen-activated protein kinase 9
30	P48736	3SD5	Phosphatidylinositol 4,5-bisphosphate 3-kinase catalytic subunit gamma isoform
31	P53779	3TTI	Mitogen-activated protein kinase 10
32	P62508	2P7A	Estrogen-related receptor gamma
33	Q9BYF1	1R4L	Angiotensin-converting enzyme 2

**Table 2:** Top twenty potential medicinal plants.

Rank	Latin name	PE_plant_	Reported bioactivity
1	*Hypericum perforatum *	1777.81	[35, 36]
2	*Ganoderma lucidum *	1560.05	[37]
3	*Holarrhena antidysenterica *	1147.22	[38, 39]
4	*Celastrus orbiculatus *	1089.44	N/A
5	*Murraya euchrestifolia *	1066.97	N/A
6	*Melia azedarach *	980.47	[40]
7	*Datura metel *	894.36	[41, 42]
8	*Ficus microcarpa *	837.65	[43]
9	*Tripterygium wilfordii *	785.30	[44]
10	*Pachysandra terminalis *	740.38	N/A
11	*Calendula officinalis *	729.77	[45]
12	*Vitis vinifera *	719.77	[46]
13	*Melia toosendan *	711.49	N/A
14	*Mangifera indica *	677.08	[47]
15	*Piper nigrum *	667.41	[48]
16	*Solanum dulcamara *	667.12	[49]
17	*Garcinia hanburyi *	641.41	N/A
18	*Momordica charantia *	632.37	[50, 51]
19	*Lantana camara *	625.64	[52]
20	*Ceriops tagal *	623.13	[53]
